# Dynamic In Vitro Gastric Digestion of Sheep Milk: Influence of Homogenization and Heat Treatment

**DOI:** 10.3390/foods10081938

**Published:** 2021-08-20

**Authors:** Zheng Pan, Aiqian Ye, Siqi Li, Anant Dave, Karl Fraser, Harjinder Singh

**Affiliations:** 1 Riddet Institute, Massey University, Private Bag 11 222, Palmerston North 4442, New Zealand; Z.Pan@massey.ac.nz (Z.P.); S.Li2@massey.ac.nz (S.L.); A.Dave@massey.ac.nz (A.D.); karl.fraser@agresearch.co.nz (K.F.); H.Singh@massey.ac.nz (H.S.); 2 AgResearch, Private Bag 11 008, Palmerston North 4442, New Zealand

**Keywords:** sheep milk, protein, fat, pepsin, homogenization, heat treatment, protein coagulation, structure, gastric digestion

## Abstract

Milk is commonly exposed to processing including homogenization and thermal treatment before consumption, and this processing could have an impact on its digestion behavior in the stomach. In this study, we investigated the in vitro gastric digestion behavior of differently processed sheep milks. The samples were raw, pasteurized (75 °C/15 s), homogenized (200/20 bar at 65 °C)–pasteurized, and homogenized–heated (95 °C/5 min) milks. The digestion was performed using a dynamic in vitro gastric digestion system, the human gastric simulator with simulated gastric fluid without gastric lipase. The pH, structure, and composition of the milks in the stomach and the emptied digesta, and the rate of protein hydrolysis were examined. Curds formed from homogenized and heated milk had much looser and more fragmented structures than those formed from unhomogenized milk; this accelerated the curd breakdown, protein digestion and promoted the release of protein, fat, and calcium from the curds into the digesta. Coalescence and flocculation of fat globules were observed during gastric digestion, and most of the fat globules were incorporated into the emptied protein/peptide particles in the homogenized milks. The study provides a better understanding of the gastric emptying and digestion of processed sheep milk under in vitro gastric conditions.

## 1. Introduction

Sheep milk is of high nutritional value and has potential for the development of nutritional and functional milk products, attracting a growing number of consumers worldwide [1]. Milk, as an important source of protein for humans, has been widely examined for its digestion behavior in both in vivo and in vitro studies [2,3,4]. The digestion of cow milk has been investigated extensively, whereas the digestion of noncow milk (i.e., sheep milk) is less studied.

Sheep milk and cow milk vary significantly in composition, physicochemical properties, and structures, which may potentially lead to different digestion behaviors within the gastrointestinal tract and the bioavailability of nutrients [1]. Jasińska [5], conducted a study to examine the hydrolysis of the casein micelles in the raw milks from 4 species (human, goat, mare, and two breeds of cow) and showed that the degrees of hydrolysis of the caseins by pepsin were 80%, 65%, 45%, 42%, and 23% for human, goat, mare, black and white cow, and red polish cow milk, respectively. Jasińska [5], attributed the differences in casein hydrolysis in the milks from different species to the different physicochemical properties and compositions of the caseins such as micellar structure and different levels of β-casein. Previous studies have also shown that the different compositions of milk proteins can result in different digestion behaviors [6,7,8]. For instance, goat milk has lower α_s1_-casein content and higher β-casein content than cow milk, and infant formulas made from goat milk formed smaller flocs of aggregated proteins and fat globules during in vitro gastric digestion, resulting in faster protein digestion in the infant formula made with goat milk than in that made with cow milk [7,9]. Sheep milk has markedly higher levels of β- and α_s2_-casein but lower levels of α_s1_-casein than cow milk, which may potentially affect its coagulation behavior and protein hydrolysis in the stomach [10]. Previous research comparing the in vitro gastric digestions of cow, goat, and sheep milks found that the curds formed from sheep skim milk had higher total solids and lower moisture contents than those formed from cow and goat skim milks because of their different chemical compositions, resulting in a firmer curd from the sheep skim milk [6].

Milk is commonly exposed to different processing treatments (i.e., pasteurization and homogenization), which leads to structural changes in its components (i.e., protein and fat). For instance, the heat treatment of milk could result in a series of protein–protein and protein–lipid interactions and changes, depending on the heat intensity level [11,12,13,14]. The homogenization of milk increases the stability of the milk fat globules because of a decrease in fat globule size and the adsorption of caseins and whey proteins onto the surface of the newly formed milk fat globules [15]. Additionally, homogenization coupled with the heat treatment of milk increases the association of denatured whey proteins with the adsorbed caseins and milk fat globule membrane (MFGM) proteins via disulfide bonds, leading to alteration of the interfacial composition of the fat globules [16]. In turn, these changes in the milk components could have an impact on the digestion behavior of milk within the gastrointestinal tract.

Roy et al. [17] investigated the effect of pasteurization on the in vitro gastric digestion of milks from cow, goat, and sheep and found that all pasteurized milks formed less integrated curds than their raw milk counterparts, resulting in a greater extent of deformation and thus higher levels of fat release into the liquid phase. However, the effect of homogenization and intensive heat treatment on sheep milk has not been investigated. There has been extensive research on the in vitro digestion of cow milk treated with more intense heat treatment. The curds formed from intensively heated cow milk were more fragmented and crumbly compared with the more cohesive curds formed from unheated or pasteurized milk, which was attributed to the differences in the structural changes in the milk components that were induced by the different processing treatments [4,18]. The content and the structure of the curds formed from homogenized cow milk during gastric digestion also showed differences compared with those formed from raw cow milk. Ye et al. [19] reported that homogenized milk formed an integrated curd but with a more porous structure than that formed from untreated whole milk in the early stage of in vitro gastric digestion, and that the curd became less integrated and was separated into several small pieces at longer digestion times. Milks treated with a combination of homogenization and heat treatment were digested more effectively than those treated with either heat treatment or homogenization alone [4,19]. For example, Ye et al. [19] investigated the effects of homogenization and heat treatment on the formation of curds during the in vitro gastric digestion of whole cow milk and observed that homogenization of the milk followed by heat treatment resulted in the formation of curds with more fragmented and crumbly structures than those formed from raw and singly homogenized whole milk. These differences in the digestion behaviors of differently processed milks suggest that they are likely to have different physiological effects (i.e., level of satiety and secretion of cholecystokinin) within the gastrointestinal tract [20]. Therefore, the effect of homogenization and intensive heat treatment on the digestion behavior of sheep milk was investigated. Egger et al. [21,22], investigated the protein hydrolysis in cow skim milk powder using a simple in vitro static digestion model and in vivo pig digestion model and found that the protein patterns at the endpoints of gastric digestion were similar in both models. The comparison of the gastric digestion behaviors of pasteurized and UHT homogenized cow milks using a rat model and an in vitro dynamic human gastric simulator was assessed by Ye et al. [18]. The formation of curds in the stomach was found in both in vivo and in vitro and the protein digestion was following a similar trend in both models [18]. It suggests that the digestion behavior of milk observed in in vitro dynamic digestion model could generate a good approximation to the in vivo results.

In this study, both untreated and processed (homogenization at 200/20 bar and 65 °C, pasteurization at 75 °C for 15 s, and heat treatment at 90 °C for 5 min) sheep milks were digested using a dynamic in vitro gastric digestion model (a human gastric simulator, HGS) to investigate the effects of homogenization, heat treatment, and the combination of homogenization and heat treatment on their gastric digestion behaviors, including coagulation, curd structure, protein digestion, and the release of protein and fat from the stomach.

## 2. Materials and Methods

### 2.1. Milk Supply and Processing Treatments

Fresh sheep milk was collected from Spring Sheep Milk Co. (Auckland, New Zealand) and Maui Milk Co., Ltd., Waikato, New Zealand, during mid-lactation; the milks collected from the two companies were mixed at a ratio of 1:1. Pasteurization of the sheep milk was carried out at 75 °C for 15 s in a pilot-scale indirect UHT plant (Alfa-Laval, Huntingwood, NSW, Australia). The homogenized milk was obtained by homogenizing raw sheep milk at 200/50 bar and 65 °C in a 2-stage valve homogenizer in the Massey University pilot plant. In the experiments, the homogenized sheep milk was pasteurized at 75 °C for 15 s to make homogenized–pasteurized (homo–past) sheep milk; the homogenized and heated (homo–heat) sheep milk was obtained by heating to reach 95 °C in the UHT plant and then transferred to a water bath for holding for 5 min. The parameters for pasteurization selected for processing of sheep milk were based on the codes of practice documents of New Zealand Food Safety Authority [23]; the parameters for homogenization were commonly used in industrial processing of milk; the heat treatment at 95°C for 5 min is commonly used in the processing of yogurt [24]. After heat treatment, these milk samples were immediately cooled to 20 °C. The average fat globule sizes (*d*_43_) of the milk samples, which were determined using a Mastersizer 2000 (Malvern Instruments Ltd., Malvern, UK), were 4.52 ± 0.14 μm and 0.62 ± 0.07 μm for the unhomogenized and homogenized sheep milks, respectively.

### 2.2. Chemicals for In Vitro Gastric Digestion

Pepsin from porcine gastric mucosa (EC 3.4.23.1; Catalogue No. P7000, Sigma Chemical Co., St. Louis, MO, USA) had an enzymatic activity of 550 units/mg solid, as tested in preliminary experiments. All other chemicals were of analytical grade and were purchased from BDH Chemicals (BDH Ltd., Poole, UK) and Sigma Chemical Co. (St. Louis, MO, USA) unless otherwise specified. All solutions were prepared using Milli-Q water purified by treatment with a Milli-Q apparatus (Millipore Corp., Bedford, MA, USA).

Simulated salivary fluid (SSF) and simulated gastric fluid (SGF) were prepared at 1.25× concentration according to the method described by Brodkorb et al. [25] with slight modifications. SSF was prepared based on the salt composition only, as described in Brodkorb et al. [25], because milk contains no starch. The SGF (pH 1.5) did not include gastric lipase because this study focused on the formation of protein curd and protein digestion induced by pepsin. CaCl_2_ (H_2_O)_2_ was added into the SSF and the SGF immediately before the digestion experiment to achieve final concentrations of 1.5 mM and 0.15 mM, respectively. Furthermore, the SSF and SGF were supplemented with water to achieve a 1× concentration before addition into the stomach chamber.

### 2.3. In Vitro Gastric Digestion

A dynamic in vitro gastric model was used to mimic the dynamic gastric digestive process. The HGS developed by Kong and Singh [26] was employed for the in vitro gastric digestion of the sheep milks. The method described in Ye et al. [19] was used for the gastric digestion with slight modifications. A 200 g sheep milk sample pre-warmed at 37 °C was firstly mixed with an amount of SSF that equaled the total solids content of the milk samples (i.e., 18.5 g of solids in the sheep milk to 18.5 g of SSF), and then transferred and warmed in the HGS at 37 °C for 2 min; a 20 mL fasting solution containing SGF (16 mL) and pepsin solution (4 mL, 10,000 units/mL, prepared in water) was added into the mixture of milk sample and SSF [27,28]; the SGF and the pepsin solution (10,000 units/mL) were then added using two separate pumps at flow rates of 2.4 mL/min and 0.6 mL/min, respectively, to achieve a 1× concentration of SGF and a pepsin activity of 2000 units/mL; the gastric emptying rate was 3.6 mL/min; the emptied digesta were removed from the bottom of the stomach chamber at 20-min intervals for accurate control of the gastric emptying. To mimic the contraction and temperature of the stomach, the contraction frequency of the HGS was set at 3 times/min and the temperature was maintained at 37 °C using a heater and a thermostat. The gastric digestion time was up to 240 min, but most of the experiments were stopped at different times to collect the coagulated milk curds for further analysis. The digesta removed from the HGS at each time interval were filtered through a mesh with a pore size diameter of 1 mm for further analysis, and the solid mass with size greater than 1 mm was put back into the HGS for further digestion. The processing and the digestion experiments were triplicated with 3 different batches of raw sheep milk.

### 2.4. pH Measurement

The pH of the mixture of milk and SSF was defined as the initial pH in the HGS. The pHs of the emptied digesta at different digestion time points refer to the pH in the HGS as the simulated gastric contraction prevented easy access into the gastric chamber for direct determination using the pH meter.

### 2.5. Weight of Curds

The content within the HGS at different digestion time points was collected and filtered through a sieve mesh with a 1-mm pore size to separate the curd and the aqueous phase. Curd larger than 1 mm was then immediately rinsed with pepsin-free SGF and weighed to obtain the weight of the curds. Subsequently, these curds were heated at 90 °C for 5 min to inactivate the pepsin and then dried at 105 °C for 24 h in a vacuum oven, so that the weight of total solids of the curd could be determined. The measurements of the curd weight were duplicated with 2 different batches of sheep milk.

### 2.6. Calcium, Protein, and Fat Content Analysis

The total calcium content of the dried curds was determined by inductively coupled plasma optical emission spectroscopy after the curd powder had been dissolved and digested by nitric and hydrochloric acids. The protein and fat contents of the curds and digesta obtained at different digestion times from different milk samples were determined using the Dumas method (AOAC 968.06) and the Mojonnier method (AACC 30-10) [29]. The protein content was calculated using a conversion factor of 6.25 for multiplication of the nitrogen content of the curds and digesta. The analyses of calcium, protein, and fat content of curds were duplicated with 2 different batches of sheep milk.

### 2.7. Protein Hydrolysis

The protein compositions of the curds and the emptied digesta were determined using tricine sodium dodecyl sulfate-polyacrylamide gel electrophoresis (tricine SDS-PAGE). The sample buffer (containing 40% glycerol, 2% SDS, 0.04% Coomassie Brilliant Blue G-250, 5% β-mercaptoethanol, and 20% 0.2 M Tris-HCl, pH 6.8) was mixed with liquid digesta to achieve a protein concentration of 1 mg/mL. For the solid curd samples, lyophilized and ground curd powder was dissolved in 1 mL of sample buffer to achieve a protein concentration of 1 mg/mL. These mixtures were then heated at 90 °C for 5 min, and 7 μL of these heated mixtures was loaded in the wells of an electrophoresis gel that was pre-made using a Mini-PROTEAN electrophoresis system. The electrophoresis gel was composed of resolving gel (16% acrylamide, glycerol, 3 M Tris-HCl buffer, pH 8.45) and 4% stacking gel (4% acrylamide, 3 M Tris-HCl buffer, pH 6.8). Two running buffers were employed to separate low-molecular-mass proteins/peptides with high resolution: an anode buffer (0.2 M Tris-HCl, pH 8.9) and a cathode buffer (0.1 M Tris base, 0.1 M tricine, and 0.1% SDS, pH 8.25). After running at a constant voltage of 120 V for around 2 h, the gel was fixed using 5% glutaraldehyde with constant gentle shaking for 25 min. The fixed gel was stained for 20 min using 0.03% Coomassie Brilliant Blue G-250 solution (0.3 g of Coomassie Brilliant Blue G-250 in 1 L of 10% glacial acetic acid solution) and then destained with destaining solution (10% glacial acetic acid solution) for 2 h to remove excess dye. The protein patterns of the gels were imaged and analyzed using a Molecular Dynamics Model PD-SI computing densitometer (Molecular Dynamics Inc., Sunnyvale, CA, USA).

### 2.8. Microstructure of Curds and Digesta

A confocal laser scanning microscope (Leica Microsystems Pty Ltd., Heidelberg, Germany) was used to study the microstructure of the digesta obtained from the digestion of the milks. The curd and digesta samples were stained and observed immediately without inactivating the pepsin after collection, according to the method described by Wang et al. [30]. The fluorescent dyes Nile Red and Fast Green were used to stain the oil (argon laser with an excitation line at 488 nm) and the protein (He–Ne laser with excitation line at 633 nm), respectively. A 200 μL aliquot of digesta was transferred into an Eppendorf tube and then mixed with 5 μL of 1.0% (*w*/*v*) Fast Green and 10 μL of 0.1% (*w*/*v*) Nile Red. The samples were stained for at least 5 min. For curd samples, an aliquot was taken using a blade and stained with 1.0% Fast Green and 0.1% Nile Red for at least 10 min. The stained samples were placed on concave confocal microscope slides (Sail; Sailing Medical-Lab Industries Co. Ltd., Suzhou, China), covered with a coverslip, and examined with ×40 and ×100 oil immersion lenses. The microstructures of digesta and curds were measured once at each time point with multiple fields.

### 2.9. Statistical Analysis

Each experiment was performed triplicates unless specified in methods using freshly prepared milk samples. Data plotting and statistical analysis were performed using GraphPad Prism 8.4.0 (GraphPad Software, San Diego, CA, USA). Statistical analysis was performed using 1-way analysis of variance and Tukey’s multiple comparison test at a significance level of *p* < 0.05. Relationships between protein release and fat release from the curds were determined using nonlinear regression analysis. The results are presented as the mean ± standard deviation.

## 3. Results and Discussion

### 3.1. Gastric pH Profile

**Figure 1:**
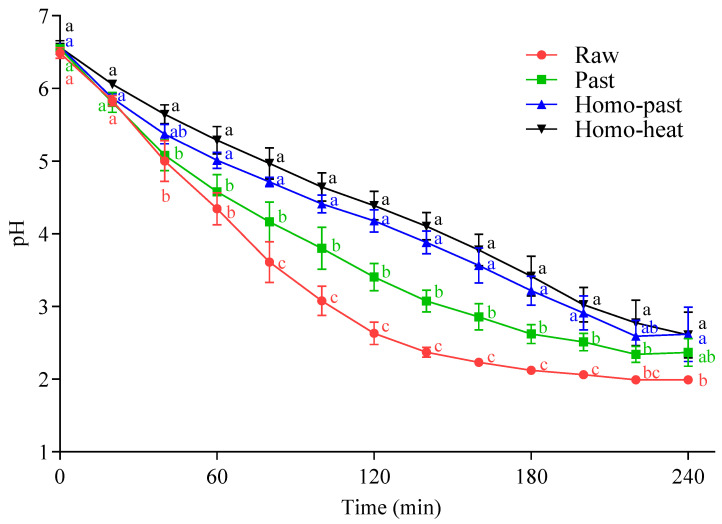
pH changes during the in vitro gastric digestion of differently processed sheep milks: ●, raw milk; ■, pasteurized (Past) milk; ▲, homogenized and pasteurized (Homo–past) milk; ▼, homogenized and heated (Homo–heat) milk. Different lowercase letters indicate significant difference (*p* < 0.05). Error bars represent standard deviations.

The pH profiles of the milk samples in the HGS are shown in Figure 1. The pHs of all milk samples decreased progressively, reaching a pH of between 2 and 3 at 240 min of digestion. However, the pHs at 80–200 min of digestion showed statistically significant differences (*p* < 0.05) among the milk samples, except for the pHs of the homo–past and homo–heat milks (*p* > 0.05). The homogenized milk samples (homo–past and homo–heat) had a markedly slower decrease in pH with the digestion time, reaching about pH 2.6 at 240 min of digestion, compared with the unhomogenized milk samples (pH 2.0 and pH 2.3 for the raw and pasteurized milks, respectively). There was also a difference in the pH-decrease patterns between the unheated and heated milk samples; the effect of stronger heating conditions on the pH profiles was more marked, resulting in a slower decrease in pH. These differences were probably related to the formation of curds with different structures in the differently treated milk samples, which might lead to different diffusion rates of molecules and ions from the SGF into and out of the curds.

### 3.2. Gastric Coagulation of Sheep Milk

**Figure 2:**
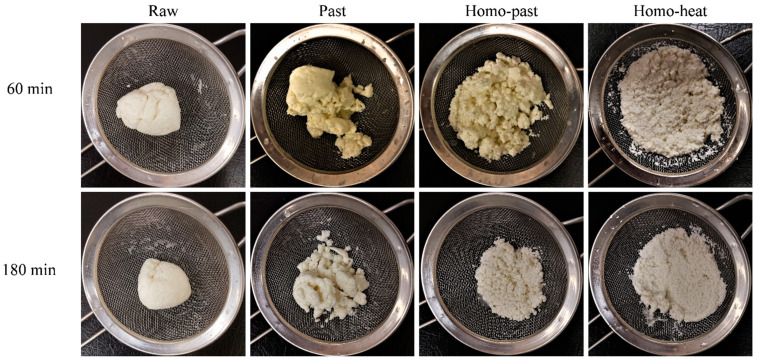
Appearance of curds collected at 60 and 180 min during the in vitro gastric digestion of raw, pasteurized (Past), homogenized and pasteurized (Homo–past), and homogenized and heated (Homo–heat) sheep milks.

The appearance of the curds formed at different digestion times from differently processed sheep milks is shown in Figure 2. In all milk samples, protein coagulation was visible immediately after the addition of the milk into the SGF (20 mL of SGF with a pepsin activity of 2000 units/mL). After 60 min of digestion, a firm curd with a smooth surface was observed in both the raw milk and the pasteurized milk; at this stage, the serum phase became clear, indicating that most of the casein micelles and fat globules were incorporated into the curd. With further digestion to 180 min, the firm curds formed from the raw milk samples became smaller but remained integrated, whereas the curds formed from the pasteurized milk samples became less integrated and were broken into several small pieces with various sizes (Figure 2). However, the curds from the homo–past and homo–heat milks appeared to be more fragmented and looser than those from the raw and pasteurized milks throughout the gastric digestion. In the homo–heat sheep milk, the curd crumbles were much looser, smaller, and more evenly fragmented than those from the homo–past milk. These results indicated that homogenization and different levels of heat treatment of sheep milk could lead to the formation of differently structured curds.

Previous studies have suggested that the formation of curds in the stomach is initially driven by the enzymatic action of pepsin on κ-casein [3,31,32]. The present study showed that the coagulation of sheep milk occurred immediately after the mixing with SGF, in which the pH in the system was greater than 6. The result was in agreement with previous findings for cow milk [3,19].

### 3.3. Microstructure of Curds

**Figure 3:**
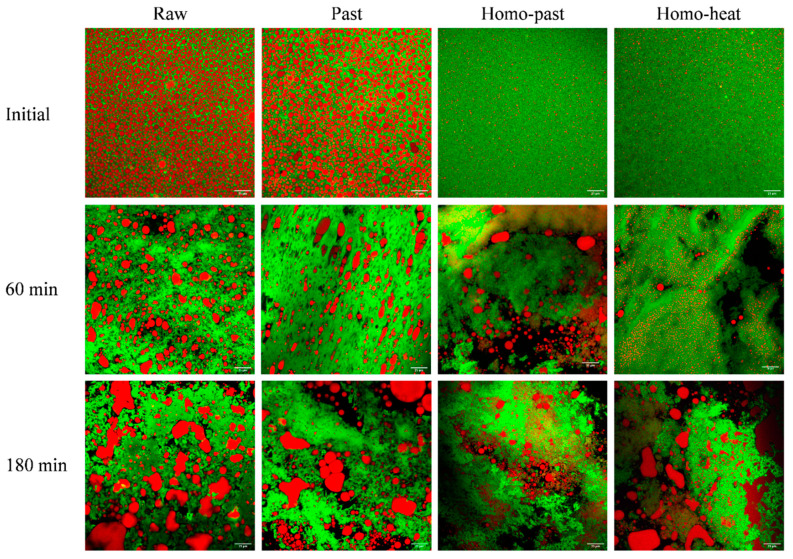
Confocal micrographs of curds obtained at 60 and 180 min during in vitro gastric digestion of raw, pasteurized (Past), homogenized and pasteurized (Homo–past), and homogenized and heated (Homo–heat) sheep milks. Red shows the fat and green shows the protein. The scale bar in all images is 25 μm.

The structures of the milk curds formed from the raw, pasteurized, homo–past, and homo–heat sheep milks at different digestion times were determined using confocal microscopy (Figure 3). For all milk samples before digestion, the fat globules were evenly distributed in the protein aqueous phase, and the fat globule size in the homogenized milks appeared to be much smaller than that in the unhomogenized milks. At 60 min of digestion, a closely-knit network of protein matrix was observed in all milk samples, and many fat globules were also found within the protein matrix. Additionally, the size of the fat globules within the curd became larger during the digestion progress, compared with that in undigested milk samples, suggesting that aggregation and/or coalescence of the fat globules occurred because of the effect of hydrolysis by pepsin on the membrane proteins surrounding the fat globules [33,34].

The small fat globules of the homo–past and homo–heat sheep milks appeared to be well embedded within the protein matrix, whereas the fat globules of the raw and pasteurized milks seemed to be entrapped within the curd without much contact with the protein network (Figure 3). The difference between the homogenized and unhomogenized sheep milk samples was probably related to the changes in the structure of the casein micelles and the interfacial protein composition of the fat globules that were caused by homogenization and heat treatment. Homogenization of milk leads to increases in the total surface area of the fat globules and the adsorption of caseins and whey proteins [15,35]. These proteins that coat the smaller globules may interact with the protein network within the curd, leading to the structural changes in the curds during the gastric digestion [4]. Further, more intense heat treatment of the homogenized sheep milk could result in a higher level of association of denatured whey proteins with the casein micelles and with MFGM proteins, which may reduce the casein–casein interactions and casein–fat interactions and thus hinder the aggregation and coagulation of milk proteins [18]. It appears that these structural changes of the fat globules and proteins that were induced by homogenization and more severe heat treatment were responsible for the formation of the differently structured curds, as observed in Figure 2.

### 3.4. Disintegration of Curds

#### 3.4.1. Weight of Curds

**Figure 4:**
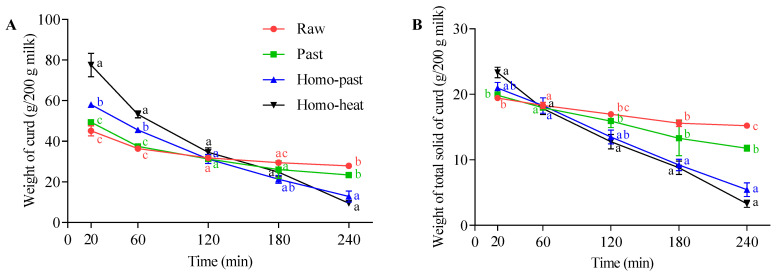
Changes in (**A**) the weight of the curds and (**B**) the weight of the total solids of the curds at different time points (20–240 min) during the in vitro gastric digestion of differently processed sheep milks: ●, raw milk; ■, pasteurized (Past) milk; ▲, homogenized and pasteurized (Homo–past) milk; ▼, homogenized and heated (Homo–heat) milk. Different lowercase letters indicate significant difference (*p* < 0.05). Error bars represent standard deviations.

The weights of the curd and the total solids of the curds in all milk samples decreased gradually throughout the gastric digestion (Figure 4A,B). The initial (20 min) weights of the curds and the total solids of the curds followed the order raw < pasteurized < homo–past < homo–heat, indicating that homogenization and more intense heating resulted in the incorporation of more milk components into the curds. This was attributed to the whey proteins that associated with the casein micelles during the heat treatment [18]. Statistical analysis showed that there were significant differences in the weights of the curds between the homogenized (homo–past and homo–heat) milks and the unhomogenized (raw and pasteurized) milks (*p* < 0.01) and between the homo–past and homo–heat milks at 20 min (*p* < 0.05), whereas there were no significant differences in the weights of the total solids of the curds (*p* > 0.05), suggesting that more moisture might be retained in the curds formed from the homogenized and heated sheep milks. This result was in agreement with previous results, which showed that curds with a looser structure contained more liquid [18,36]. Thus, a curd with higher moisture content might contain more SGF and pepsin. With further digestion, the weights of the curds and the total solids of the curds decreased much faster in the homo–past and homo–heat sheep milks than in the raw and pasteurized sheep milks. Significant differences between each time point in the weights of the curds and the total solids were observed in the homo–past and homo–heat sheep milks (*p* < 0.05) but not in the raw and pasteurized sheep milks. At 240 min of digestion, both the weight of the curds and the weight of the total solids showed a reverse order compared with their initial weights: raw > pasteurized > homo–past > homo–heat. These results suggested that pasteurization alone of sheep milk did not have much impact on the breakdown of the curd compared with the raw sheep milk; pasteurization combined with homogenization significantly accelerated the disintegration of the sheep milk curds; more intense heat treatment of the homogenized sheep milk did not further impact the breakdown of the curds compared with the homo–past sheep milk but resulted in a significantly higher weight of the curds at 20 min.

#### 3.4.2. Changes in Protein, Fat, and Calcium Contents in the Curd

**Figure 5:**
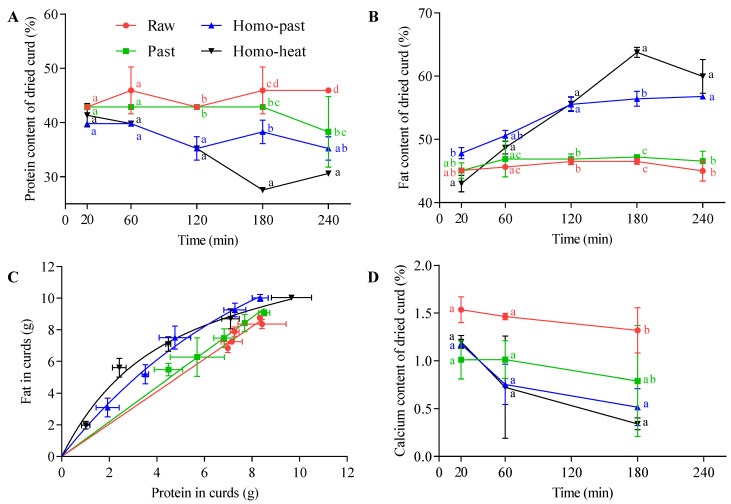
Changes in (**A**) the protein content of the dried curds and (**B**) the fat content of the dried curds, (**C**) the relationship between the amounts of fat and protein in the curds, and (**D**) the calcium content of the dried curds at different time points (20–240 min) during the in vitro gastric digestion of differently processed sheep milks: *●*, raw milk; *■*, pasteurized (Past) milk; *▲*, homogenized and pasteurized (Homo–past) milk; *▼*, homogenized and heated (Homo–heat) milk. Different lowercase letters indicate significant difference (*p* < 0.05). Error bars represent standard deviations.

Figure 5A shows the protein content of the curds as the function of digestion time in the 4 different sheep milks. The protein contents of the raw and pasteurized milks remained nearly constant at around 43% during the digestion period, whereas those of the homo–past and homo–heat sheep milks showed a decreasing trend as the digestion progressed, especially after 60 min. For the homo–past milk, the protein content of the curds decreased in the first 120 min, after which it remained roughly unchanged at around 36% until the end of digestion. The decreasing trend for the protein content of the homo–heat milk continued to 180 min (from ~40–~30%). The fat content of the curds is shown in Figure 5B. Similar to the protein content, there were no obvious changes in the fat content of the curds of the unhomogenized milks throughout the gastric digestion, with a fat content of ~45% for the raw milk and of ~46% for the pasteurized milk. However, the fat contents of the curds of the homogenized milks showed an opposite trend compared with their protein counterparts. The fat contents of the curds increased for 120 min and 180 min of digestion for the homo–past milk (from 47.8–55.6%) and the homo–heat milk (from 43–63.8%), respectively, after which the level remained almost unchanged.

The relationships between the amounts of protein and fat in the curds are shown in Figure 5C. The slope of the regression line for the raw and pasteurized sheep milks was close to 1, indicating a strong correlation between fat and protein in the curds at different time points. Similar results have been reported for the gastric digestion of unheated and heated cow milks [36]; the slopes of the regression lines for the fat–protein profiles of the curds formed from these milk samples were close to 1. This indicates that the fat was evenly distributed in the curds and was released from the curds in equal proportion to the protein. However, a nonlinear correlation between the fat and protein in the curds was found for the homo–past and homo–heat sheep milks, suggesting that fat and protein had different rates of release from the curd. The amounts of fat and protein in the homo–past and homo–heat sheep milks were significantly lower than those in the raw and pasteurized sheep milks at 240 min of digestion (the leftmost points in Figure 5C) (*p* < 0.05), indicating that the release of fat and protein occurred more rapidly in homogenized sheep milk than in unhomogenized sheep milk.

The homo–past and homo–heat sheep milks had higher initial weights of the fat in the curds than the raw and pasteurized sheep milks (the rightmost point in Figure 5C). Previous studies have suggested that newly formed fat globules in milk after homogenization are covered by caseins and whey proteins and are able to act as pseudo-protein particles that can interact with the protein phase during coagulation, becoming an integral part of the protein matrix and thus resulting in higher fat contents of the curds [37,38]. The microstructures of the curds (Figure 3) also confirmed these findings. Further, the inclusion of smaller fat globules in the protein matrix can soften the casein network because the homogeneously distributed small fat globules in the protein network result in a larger intermicellar distance and thus hinder the fusion and syneresis of the casein matrix [35,39]. It should be noted that the gastric lipase was not included in SGF in this study because we mainly focused on the investigation of the formation of protein curd and protein digestion under gastric conditions. Previous studies have shown that the gastric lipolysis represents 10% to 30% of total lipid digestion throughout the gastrointestinal tract [2], but the reciprocal effects of gastric lipase and pepsin on the milk digestion behavior are unclear. The inclusion of gastric lipase in the gastric digestion of milk may have an impact on the breakdown of curds and the release of fat from the curds, further investigations need to be taken in the future.

The calcium contents of the dried curds obtained from the 4 different types of sheep milk are presented in Figure 5D. The calcium content in all curds decreased with increasing digestion time, but the rates of decrease were different between the unhomogenized and homogenized milks. The calcium contents of the dried curds in the raw and pasteurized milks decreased steadily over the digestion time, whereas those in the homo–past and homo–heat milks decreased rapidly in the first 60 min and then slowly in the following 120 min. These results suggest that calcium was gradually released from the curd to the liquid phase as the digestion time increased. It has been suggested that bound calcium solubilizes when the pH of the curds decreases to below 5.6, resulting in the release of the solubilized calcium into the liquid phase with the progress of the digestion [40]. The fast release of calcium from the curds of the homo–past and homo–heat sheep milks could be attributed to the faster diffusion of the acid (SGF) into the curds because of their fractured and looser structure (Figure 2). In comparison, the lower rate of calcium release from the curds of the raw and pasteurized sheep milks was due to the formation of an integrated curd surface barrier (Figure 2) that probably impeded the flowing SGF.

These results suggest that the release of the curd contents (protein, fat, and calcium) was markedly dependent on the structure of the curds. The curd formed from the raw and pasteurized sheep milks was more integrated and firmer, which may have impeded the diffusion of the SGF and pepsin into the curds, resulting in a slower breakdown of the curd structure and a slower release of the curd contents. In contrast, the looser and crumbled structures of the curds of the homo–past and homo–heat sheep milks were easily deformed by the physical forces of gastric contraction, leading to a faster release from the curds. These results are in line with previous results on cow milk reported by Ye et al. [18], who showed that the homogenization of cow milk followed by heat treatment made the structure of the curds more open, leading to rapid loss of total solids and rapid release of fat from the curds during gastric digestion. The faster release of calcium and fat from curds of homogenized sheep milk may lead to faster fat digestion and thus enhance uptake of calcium and fat in the small intestine.

### 3.5. SDS-PAGE Protein Patterns of Curds

**Figure 6:**
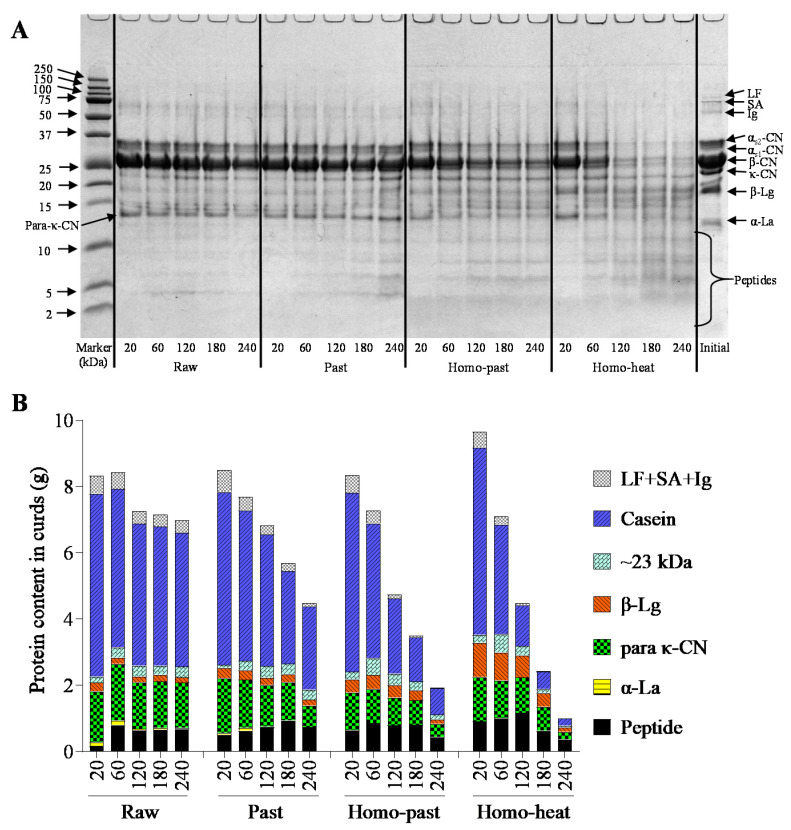
(**A**) Reducing tricine SDS-PAGE patterns and (**B**) protein contents of curds obtained from raw, pasteurized (Past), homogenized and pasteurized (Homo–past), and homogenized and heated (Homo–heat) sheep milks. The numbers (20, 60, 120, 180, 240) refer to different gastric emptying times (min). Initial refers to before digestion. The protein concentration in each sample for the tricine SDS-PAGE was 1 mg/mL.

The protein hydrolysis by pepsin in the curds was determined using tricine SDS-PAGE (Figure 6A). The amount of each individual protein in the curds is shown in Figure 6B. The κ-casein (κ-CN) band disappeared and 2 new bands at ~23 kDa (macropeptides) and 14 kDa (para-κ-CN) appeared in all samples. Interestingly, unlike the α-lactalbumin (α-La) band, which disappeared in all curd samples, a band corresponding to β-lactoglobulin (β-Lg) was observed throughout the digestion in all types of sheep milk. At 20 min, the amounts of β-Lg in the curds were raw (~0.26 g) < pasteurized (~0.31 g) < homo–past (~0.4 g) < homo–heat (~1.03 g) (Figure 6B), indicating that greater heat treatment incorporated more β-Lg into the curds. The small amount of whey proteins observed in the curds of the raw, pasteurized, and homo–past milk samples could have been caused by the entrapment of whey proteins during the formation of the curds; they could be expelled from the curds and hydrolyzed gradually as the digestion progressed [17,36]. The markedly higher β-Lg content observed in the curd formed from the homo–heat milk was due to the higher level of denatured whey proteins associated with the casein micelles and the MFGM proteins [12,15].

The changes in the intensities of all intact protein bands, including αs-CN, β-CN, and β-Lg, showed marked differences among the sheep milk samples. The intensities of these intact protein bands decreased slowly in the unhomogenized milks with increasing digestion time, but faded away much faster in the homogenized milks (Figure 6A). Bands corresponding to peptides were detected in all milk samples after 60 min of digestion, with the homogenized milks having more intense peptide bands than the unhomogenized milks. This indicates that the homogenization of sheep milk followed by heat processing led to a greater digestibility of proteins during gastric digestion. Faster degradation of proteins has been shown to be caused by the looser and crumbly structure of curds, which allows pepsin to diffuse into the curds rapidly and to hydrolyze the proteins [19]. These findings further support the faster breakdown of the curds and the release of fat and calcium from the curds in the homogenized and heated sheep milk because of the faster hydrolysis of proteins by pepsin, as observed earlier (Figure 4 and Figure 5).

### 3.6. Protein and Fat Contents of Emptied Digesta

**Figure 7:**
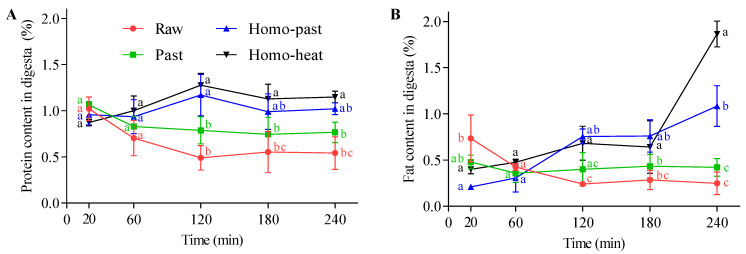
(**A)** Protein and (**B**) fat contents in the digesta emptied at different time points (0–240 min) during the in vitro gastric digestion of differently processed sheep milks: ●, raw milk; ■, pasteurized (Past) milk; ▲, homogenized and pasteurized (Homo–past) milk; ▼, homogenized and heated (Homo–heat) milk. Different lowercase letters indicate significant difference (*p* < 0.05). Error bars represent standard deviations.

The protein contents of the digesta emptied from the stomach at different digestion times from the different milk samples are shown in Figure 7A. The protein contents of the unhomogenized milks decreased gradually during 120 min of digestion, after which they remained roughly constant at ~0.41% for raw milk and ~0.69% for pasteurized milk towards the end of the digestion. However, the homo–past and homo–heat sheep milks showed the opposite trend, with the protein contents of the digesta initially increasing in the first 120 min of digestion, with little change on prolonged digestion. At digestion times of longer than 120 min, the protein contents of the digesta of the homo–past and homo–heat sheep milks were significantly higher than those of the raw and pasteurized milks (*p* < 0.05). The fat contents of the digesta are shown in Figure 7B. Those in the raw and pasteurized sheep milks decreased slightly during the first 120 min of digestion and then stayed almost unchanged on prolonged digestion, whereas the homo–past and homo–heat sheep milks showed an increasing trend over the whole digestion.

The changes in the protein contents of the digesta were in reasonable agreement with the release of protein from the curds (Figure 5). The rapid release of protein from the curds of the homo–past and homo–heat sheep milks (Figure 5) resulted in higher protein contents in the gastric chyme, thus leading to increased protein contents in the emptied digesta. In contrast, the slowly digested protein in the curds of the raw and pasteurized sheep milks could have resulted in lower levels of protein in the emptied digesta because of the dilution caused by the continuous addition of SGF. For the fat content, the trends were similar for the curds (Figure 5B) and the digesta (Figure 7B), which showed that the fat content increased throughout the digestion progress in the homo–past and homo–heat sheep milks, whereas it changed little in the raw and pasteurized sheep milks. The continuous increases in the fat contents of the emptied digesta for the homo–past and homo–heat sheep milks may have been caused by the phase separation between the water and oil phases in the stomach. Mulet-Cabero et al. [4] found that the fat content at the end of digestion was generally higher in homogenized milk than in unhomogenized milk, as a cream layer formed at longer digestion times. Thus, the cream layer could remain in the stomach until the end of digestion, resulting in increases in the fat contents of both the curds and the digesta of the homo–past and homo–heat sheep milks at longer digestion times.

### 3.7. SDS-PAGE Protein Patterns of Digesta

**Figure 8:**
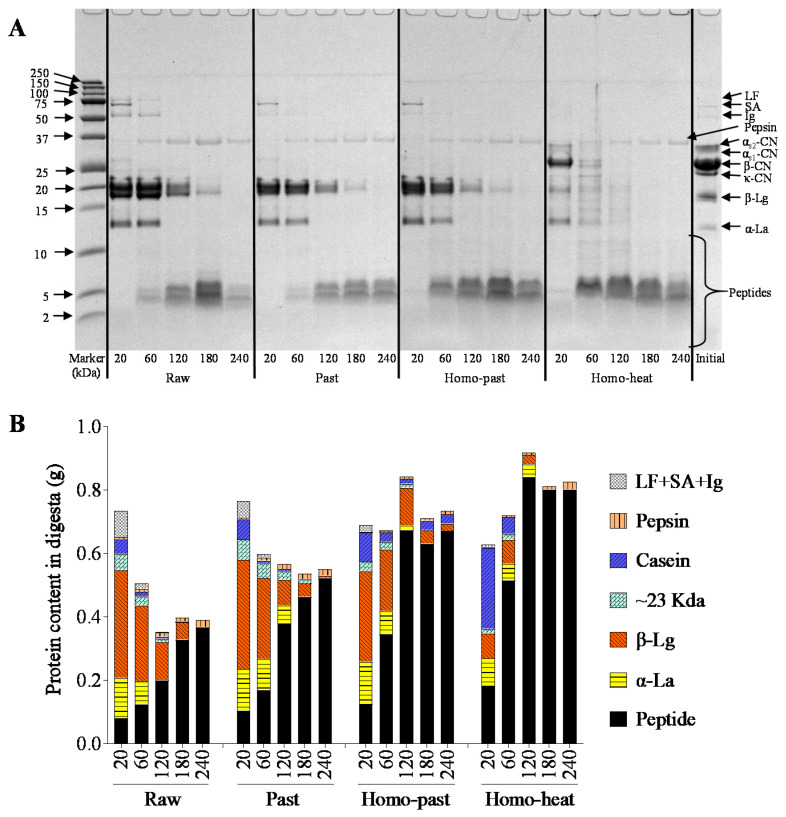
(**A**) Reducing tricine SDS-PAGE patterns and (**B**) protein contents of the digesta obtained from raw, pasteurized (Past), homogenized and pasteurized (Homo–past), and homogenized and heated (Homo–heat) sheep milks. The numbers (20, 60, 120, 180, 240) refer to different gastric emptying times (min). Initial refers to before digestion. The protein concentration in each sample for tricine SDS-PAGE was 1 mg/mL.

Figure 8A shows the protein patterns of the digesta obtained from the differently processed sheep milks. Faint intact casein bands were present at 20 min of digestion but disappeared after 60 min of digestion in the raw, pasteurized, and homo–past sheep milk samples, and bands corresponding to peptides (between 2 and 10 kDa) appeared concomitantly. This finding is consistent with previous studies, which reported that the casein bands in the digesta could be due to the delivery of very fine casein particles to the digesta when the curd lost its mass, and that increasing amounts of peptides were evacuated from the stomach because of hydrolysis of the caseins [17,19,41]. In contrast, the homo–heat milk samples showed more conspicuous intact casein bands in the digesta, the intensities of which were significantly higher than for the raw, pasteurized, and homo–past milk samples (*p* < 0.01) (Figure 8B). The difference can be attributed to the looser and crumblier structure of the curds formed from the homo–heat milk, leading to the delivery of crumbly particles (containing mostly caseins) smaller than 1 mm to the small intestine.

Differences were also found for the bands at ~14, 18, and 23 kDa, which correspond to α-La, β-Lg, and macropeptides, respectively. Within the first 120 min of digestion, there were low intensities of these bands (especially β-Lg) in the homo–heat sheep milk, whereas markedly higher intensities of these bands were observed in the other three types of sheep milk (Figure 8A). About 0.32, 0.34, 0.28, and 0.08 g of β-Lg were detected in the digesta obtained at 20 min for the raw, pasteurized, homo–past, and homo–heat milks, respectively (Figure 8B). The lower β-Lg content in the digesta of the homo–heat milk was attributed to the higher amounts of β-Lg that were incorporated in the curds (Figure 6) because of their association with the casein micelles after more intense heat treatment [42]. The β-Lg and α-La bands in all types of milk faded away gradually during the digestion and disappeared in the digesta emptied at 240 and 120 min, respectively, which is in agreement with the previous findings of Roy et al. [17]. The decreased intensities of the β-Lg and α-La bands could have been due to the dilution by the continuous addition of SGF and the hydrolysis by pepsin when the pH was less than 4 [43].

These results suggested that, in the raw, pasteurized, and homo–past sheep milks, the protein emptied from the stomach was composed mainly of whey proteins in the early stages of digestion and consisted mainly of peptides at longer digestion times because of the digestion of protein by pepsin. In comparison, the protein in the homo–heat sheep milk was digested faster, leading to a predominant content of peptides in the digesta from the beginning of digestion.

### 3.8. Microstructure of Digesta

**Figure 9:**
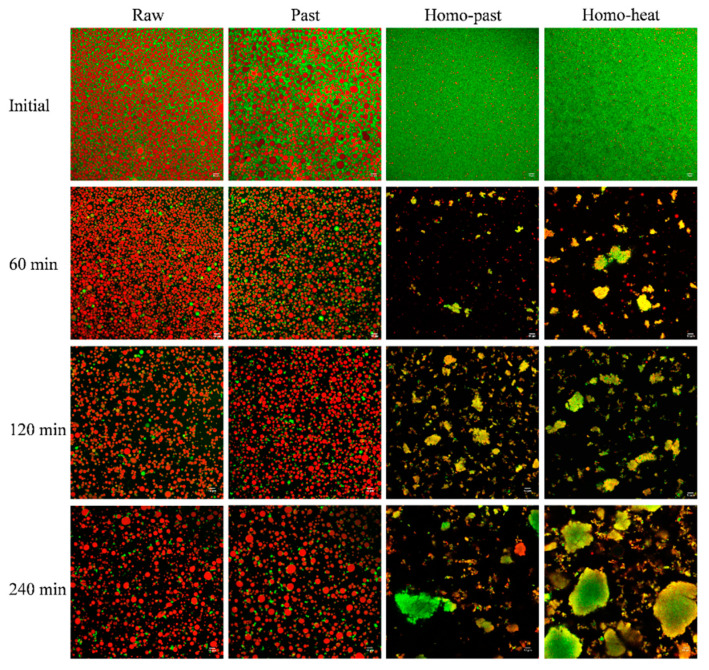
Confocal micrographs of digesta obtained at different time points (0–240 min) during in vitro gastric digestion of raw, pasteurized (Past), homogenized and pasteurized (Homo–past), and homogenized and heated (Homo–heat) sheep milks. Red shows the fat and green shows the protein. The scale bar in all images is 10 μm.

The microstructures of the fat and protein in the digesta were observed using confocal microscopy (Figure 9). There were large amounts of fat globules in the digesta of the raw and pasteurized sheep milks throughout the gastric digestion; the fat globule size appeared to remain unchanged during the first 120 min of gastric digestion but increased slightly at 240 min of gastric digestion. This is consistent with the previous report of Roy et al. [17], who also found that there were a few larger fat globules in the digesta of raw and pasteurized sheep milks at 90–240 min, which was caused by their coalescence after the MFGM proteins had been hydrolyzed by pepsin. Small-sized fat globules were found in the digesta of the homo–past and homo–heat sheep milks at 60 min of digestion, accompanied by some protein/peptide particles. There was an increasing number of protein/peptide particles with various sizes in the digesta of the homo–past and homo–heat sheep milks when the gastric digestion time was extended beyond 120 min. Interestingly, the small fat globules were still well embedded in the protein/peptide particles in the digesta of the homo–past and homo–heat sheep milks at digestion times of longer than 120 min. In agreement with the present results, a previous study demonstrated that the peptides resulting from the hydrolysis of protein by pepsin formed a new surface layer on the fat globules, which was unable to create strong interfacial layers with sufficient electrostatic repulsion and steric barriers, thus resulting in flocculation of fat globules via protein–peptide or peptide–peptide interactions [34]. Moreover, no individual fat globules were observed in the digesta of the homo–past and homo–heat sheep milks at digestion times longer than 120 min, suggesting that all fat globules had been incorporated into the protein/peptide particles. However, the incorporation of fat globules into protein/peptide particles may change the density of the particles, leading to the creaming of the less dense particles. This may support the occurrence of phase separation in the stomach, as mentioned earlier.

## 4. Conclusions

This study demonstrated the effect of heat treatment and homogenization on the dynamic in vitro gastric digestion of sheep milk. All milk samples formed structured curds in the stomach. The curds formed from the homogenized milks had a much looser and fractured structure than those formed from the unhomogenized milks because of the inclusion of smaller fat globules into the curd; the homogenization of sheep milk followed by heating at 90 °C for 5 min resulted in crumblier curds compared with homogenization coupled with pasteurization because of the incorporation of more whey proteins into the curd. The differently structured curds led to different rates of disintegration of the curds and of protein hydrolysis by pepsin, and thus impacted the gastric emptying rate. The relative rates of fat release from the curds to the emptied digesta were dependent on whether or not the sheep milk was homogenized. The release of protein, fat, and calcium from the curds occurred at much faster rates in the homogenized sheep milks than in the unhomogenized sheep milks, leading to higher fat and protein contents in the emptied digesta of the homogenized sheep milks. Flocculation of the fat globules was observed in the digesta of the homogenized sheep milks, and most of the fat globules were incorporated into the protein/peptide particles. These findings in the present study suggest that raw and pasteurized sheep milk could give persistent satiety because of its longer gastric emptying time, which could be suitable for the overweight population. In contrast, the homogenized and intensely heated sheep milk would be beneficial for elderly individuals and athletes by enhancing muscle building because of its fast protein digestion.

## Data Availability

The datasets used and/or analyzed during the current study are available from the corresponding author on request.
